# Supplementary material for: Thyroid Collision Tumors: The Presence of the Medullary Thyroid Carcinoma Component Negatively Influences the Prognosis
Source: Diagnostics (Basel). 2023 Jan 12;13(2):285. doi: 10.3390/diagnostics13020285 (PMC9857660; doi:10.3390/diagnostics13020285)
Supplement: Supplementary file 1 [file diagnostics-13-00285-s001.zip › diagnostics-2129266-supplementary.pdf]

Supplementary materials

Table S1. Pathological characteristics of TCTs' MTC components in relation to the histopathological prognostic features.

Pathological characteristics	Tumor focality		p-value	mETE		p-value	LVI		p-value	LNM		p-value
	U (#20)	M (#3)		P (#8)	A (#15)		P (#11)	A (#12)		P (#7)	A (#8)	
**Sex**												
Male	4(20%)	3(100%)	**0.020**	3(37.5%)	4(26.7%)	0.657	5(45.5%)	2(16.7%)	0.193	3(42.9%)	0(0%)	0.077
Female	16(80%)	0(0%)		5(62.5%)	11(73.35)		6(54.5%)	10(83.3%)		4(57.1%)	8(100%)	
**Tumor size (mm)**												
< 10 mm	5(25%)	2(66.7%)		1(12.5%)	6(40%)		1(9.1%)	6(50%)		0(0%)	4(50%)	
10 - 40 mm	9(45%)	0(0%)	0.253	2(25%)	7(46.7%)	0.107	3(27.3%)	6(50%)	**0.002**	2(28.6%)	4(50%)	**0.006**
> 40 mm	6(30%)	1(33.3%)		5(62.5%)	2(13.3%)		7(63.6%)	0(0%)		5(71.4%)	0(0%)	
**Amyloid**												
Present	12(60%)	1(33.3%)	0.560	6(75%)	7(46.7%)	0.379	9(81.8%)	4(33.3%)	**0.036**	6(85.7%)	3(37.5%)	0.119
Absent	8(40%)	2(66.7%)		2(25%)	8(53.3%)		2(18.2%)	8(66.7%)		1(14.3%)	5(62.5%)	
**pT category**												
T1	12(60%)	2(66.7%)		2(25%)	12(80%)		3(27.3%)	11(91.7%)		1(14.3%)	7(87.5%)	**0.005**
T2	2(10%)	0(0%)	1.000	1(12.5%)	1(6.7%)	**0.025**	1(9.1%)	1(8.3%)	**0.001**	1(14.3%)	1(12.5%)	
T3	5(25%)	1(33.3%)		4(50%)	2(13.3%)		6(54.5%)	0(0%)		4(57.1%)	0(0%)	
T4	1(5%)	0(0%)		1(12.5%)	0(0%)		1(9.1%)	0(0%)		1(14.3%)	0(0%)	
**AJCC stage**												
I	11(55%)	2(66.7%)		2(25%)	11(73.3%)		2(18.2%)	11(91.7%)		0(0%)	7(87.5%)	
II	2(10%)	1(33.3%)		1(12.5%)	2(13.3%)		2(18.2%)	1(8.3%)		0(0%)	1(12.5%	
III	2(10%)	0(0%)	0.618	1(12.5%)	1(6.7%)	**0.044**	2(18.2%)	0(0%)	**0.000**	2(28.6%)	0(0%)	**0.000**
IV	5(25%)	0(0%)		4(50%)	1(6.7%)		5(45.5%)	0(0%)		5(71.4%)	0(0%)	

*p-value <0.05 was considered to be statistically significant
Abbreviations: LVI, lymphovascular invasion; LNM, lymph node metastasis; mETE, microscopic extrathyroidal extension; P, present; A, absent; U, unifocal; M, multifocal; #, number of cases.

Table S2. Pathological characteristics of TCTs' PTC components in relation to the histopathological prognostic features

Pathological characteristics	Focality of the tumor		p-value	mETE		p-value	LVI		p-value
	U (#17)	M (#6)		P (#3)	A (#20)		P (#3)	A (#20)	
**Tumor size (mm)**									
< 10 mm	16(94.1%)	4(66.7%)	0.155	1(33.3%)	19(95%)	**0.034**	1(33.3%)	19(95 %)	**0.034**
10 - 40 mm	1(59%)	2(33.3%)		2(66.7%)	1(5%)		2(66.7%)	1(5%)	
**Growth pattern**									
Conventional	11(64.7%)	0(0%)	**0.014**	2(66.7%)	9(45%)	0.59	2(66.7%)	9(45%)	0.590
Follicular	6(35.3%)	6(100%)		1(33.3%)	11(55%)		1(33.3%)	11(55 %)	
**pT category**									
T1	16(94.1%)	4(66.7%)	0.155	1(33.3%)	19(95%)	**0.034**	1(33.3%)	19(95 %)	**0.034**
T2	1(5.9%)	2(33.3%)		2(66.7%)	1(5%)		2(66.7%)	1(5%)	
**AJCC stage**									
I	17(100%)	4(66.7%)	0.059	2(66.7%)	19(95%)	0.249	2(66.7%)	19(95 %)	0.249
II	0(0%)	2(33.3%)		1(33.3%)	1(5%)		1(33.3%)	1(5%)	
**Coexisting thyroid pathology**									
Colloid goiter	10(58.8%)	4(66.7%)	0.100	2(66.7%)	12(60%)	1.000	1(33.3%)	13(65 %)	0.190
Nodular goiter	6(35.3%)	0(0%)		1(33.3%)	5(25%)		2(66.7%)	4(20%)	
Hashimoto thyroiditis	1(5.9%)	2(33.3%)		0(0%)	3(15%)		0(0%)	3(15%)	

*p-value <0.05 was considered to be statistically significant

Abbreviations: RTL right thyroid lobe, LTL left thyroid lobe, LVI lymphovascular invasion, mETE microscopic extrathyroidal extension, P present, A absent, U unifocal, M multifocal, # - number of cases.

Table S3. Pathological characteristics of PTC-only tumors in relation to the histopathological prognostic features

Pathological characteristics	Focality of the tumor		p-value	LVI		p-value
	U (#15)	M (#8)		P (#6)	A (#17)	
**Age at diagnosis**						
<55 yo	4(26.7%)	2(25%)	1.000	4(66.7%)	2(11.8%)	**0.021**
>55 yo	11(73.3%)	6(75%)		2(33.3%)	15(88.2%)	
**Sex**						
Male	0(0%)	2(25%)	0.111	1(16.7%)	1(5.9%)	0.462
Female	15(100%)	6(75%)		5(83.3%)	16(94.1%)	
**Tumor size (mm)**						
< 10 mm	13(86.7%)	6(75%)	0.589	2(33.3%)	17(100%)	**0.002**
10 - 40 mm	2(13.3%)	2(25%)		4(66.7%)	0(0%)	
**Thyroid location**						
RTL	9(60%)	3(37.5%)	0.360	3(50%)	9(52.9%)	1.000
LTL	6(40%)	4(50%)		3(50%)	7(41.2%)	
Isthmus	0(0%)	1(12.5%)		0(0%)	1(5.9%)	
**Growth pattern**						
Conventional	8(53.3%)	6(75%)	0.400	6(100%)	8(47.1%)	**0.048**
Follicular	7(46.7%)	2(25%)		0(0%)	9(52.9%)	
**pT category**						
T1	14(93.3%)	6(75%)	0.269	3(50%)	17(100%)	**0.011**
T2	1(6.7%)	2(25%)		3(50%)	0(0%)	

*p-value <0.05 was considered to be statistically significant

Abbreviations: RTL right thyroid lobe, LTL left thyroid lobe, LVI lymphovascular invasion, P present, A absent, U unifocal, M multifocal, # - number of cases.

Table S4. Pathological characteristics of the TCTs' PTC component compared to PTC-only tumors

		Mean value	t-test value	degrees of freedom (df)	p-value
**Age at diagnosis** (years)	PTCc	61.700	0.748	44.000	0.458
	PTC-only	59.740			
**Sex** (M/F)	PTCc	1.300	1.890	36.466	0.67
	PTC-only	1.090			
**Diameter** (mm)	PTCc	6.174	-0.889	44.000	0.379
	PTC-only	8.483			
**Thyroid site** (LTL/RTL/Isthmus)	PTCc	2.430	-0.222	44.000	0.825
	PTC-only	2.480			
**Growth pattern** (Conventional/Follicular)	PTCc	1.520	0.876	44.000	0.386
	PTC-only	1.390			
**Focality of the tumor** (Unifocal/Multifocal)	PTCc	1.740	0.630	44.000	0.532
	PTC-only	1.650			
**mETE** (Present/Absent)	PTCc	1.130	1.817	22.000	0.083
	PTC-only	1.000			
**LVI** (Present/Absent)	PTCc	1.130	-1.106	41.229	0.275
	PTC-only	1.260			
**PNI** (Present/Absent)	PTCc	1.040	0.000	44.000	1.000
	PTC-only	1.040			
**Lymph node metastasis** (Present/Absent)	PTCc	1.200	-0.765	25.000	0.452
	PTC-only	1.330			
**pathologic T category** (T1/T2)	PTCc	1.130	0.000	44.000	1.000

	PTC-only	1.130			
**AJCC stage** (I/II)	PTCc	1.090	0.000	44.000	1.000
	PTC-only	1.090			

*p-value <0.05 was considered to be statistically significant

Abbreviations: M male, F female, LTL left thyroid lobe, RTL right thyroid lobe, mETE microscopic extrathyroidal extension, LVI lymphovascular invasion, PNI perineural invasion, PTCc papillary thyroid carcinoma component, PTC-only papillary thyroid carcinoma-only.
